# Analysis of Amino Acid Patterns With Nutrition Regimens in Preterm Infants With Extrauterine Growth Retardation

**DOI:** 10.3389/fped.2020.00184

**Published:** 2020-04-28

**Authors:** Li Wang, Danyang Liu, Haiqing Shen, Ying Wang, Lianshu Han, Zhenjuan He

**Affiliations:** ^1^Department of Neonatology, Xinhua Hospital, Shanghai Jiao Tong University School of Medicine, Shanghai, China; ^2^Department of Clinical Nutrition, Xinhua Hospital, Shanghai Jiao Tong University School of Medicine, Shanghai, China; ^3^Department of Pediatric Endocrine and Metabolic Diseases Laboratory, Shanghai Institute for Pediatric Research, Xinhua Hospital, Shanghai Jiao Tong University School of Medicine, Shanghai, China; ^4^Department of Perinatal Research Laboratory, Shanghai Institute for Pediatric Research, Xinhua Hospital, Shanghai Jiao Tong University School of Medicine, Shanghai, China

**Keywords:** preterm infant, extrauterine growth retardation, nutrition, amino acid, gas chromatography-mass spectrometry

## Abstract

**Background:** Amino acid (AA) metabolic patterns have emerged as an analytical technique to characterize biomarkers compromising normal growth and elucidate underlying nutritional exposure. This study aimed to identify AA metabolites most likely associated with poor growth and examine the association between AA metabolites and nutrition regimens in preterm infants during transition from parenteral nutrition (PN) to enteral nutrition (EN), using gas chromatography-mass spectrometry (GC-MS).

**Methods:** This observational cohort study was conducted in infants born at <32 weeks' gestation with birth weight of <1,500 g. The outcome of extrauterine growth retardation (EUGR) based on whether the weight was <10th percentile for post-menstrual age, was evaluated when full EN reached. Samples were collected at four sampling points according to nutritional status. AA profiles in dried sampling point spots (DBS) were quantified using GC-MS; and were compared simultaneously. The correlation of AA concentration with growth and nutritional parameters was examined using multivariate analysis.

**Results:** We identified 40 eligible infants: 20 in the EUGR group and 20 in the non-EUGR group. AA deficiency progressively emerged during the transition. Lower concentrations of four AAs, including citrulline (Cit), were associated with increased risk of EUGR when adjusted for gestational age, birth weight z-score, age when trophic EN was started, as well as average energy and protein intakes in synchronous nutritional period. Moreover, a lower Cit concentration was positively correlated with the compromised protein and energy deficits in EN during early transition.

**Conclusion:** A low Cit concentration during transition from PN to full EN should be noticed by the clinician to more closely examine nutrition practices to prevent EUGR.

## Introduction

Preterm infants are at a high risk of extrauterine growth retardation (EUGR) in the early months of life, and this is significantly associated with adverse neurodevelopmental outcomes during childhood (1–3). Protein intake has been implicated repeatedly as the limiting factor for growth (4). Increased protein and energy intake can improve postnatal growth (5, 6) and ameliorate growth restriction. Study on early nutrition of preterm infants showed that increased protein intake generates better gains in protein balance, as well as higher risk of excessive amino acid (AA) oxidation directly with supply (7) Recent studies have revealed potential relationships between perinatal growth and AA patterns. These studies catalog the metabolic changes induced by nutrition in infants and their potential impact to later-life development (8–10). It is crucial to understand the association between AA metabolism and nutrition of premature infants. If we could correct their metabolic status timely and provide reasonable nutritional support, the prognosis of preterm infants might be greatly improved (11).

AA concentrations are biomarkers of protein metabolism, and examining their patterns in preterm infants may disclose metabolic changes associated with particular conditions (12, 13). To date, normative values of AA concentrations in plasma and urine have been referred in adults, infants, and older children (13). However, there are limited data mentioning developmental changes of AA concentrations in preterm infants. The nutrition course of most preterm infants involves phases of parenteral nutrition (PN), enteral nutrition (EN) and the transitional period in between (14). Miller et al. (14) reported that the transitional period is consistent with decreased protein intake and is most likely responsible for limiting growth. However, it is unclear whether AA profiles are influenced by nutrient phases. Establishing AA profiles in preterm infants would allow evaluating their nutritional status and indicating important health outcomes for growth and neurodevelopment (13). In this study, Forty preterm infants were enrolled and divided into an EUGR group and non-EUGR group. AA concentrations were dynamically examined to analyze AA metabolic changes, and correlations between metabolite values, gestational age (GA), birth weight (BW), maternal and postnatal history, age at sample collection, nutrient intakes, and clinical and growth parameters were identified. This study thus aimed (1) to compare AA metabolites between the EUGR and non-EUGR infants, (2) to identify the correlation of AA metabolites to EUGR, and (3) to examine associations between energy–protein intakes and AA patterns.

## Methods

### Study Population

This prospective observational study was conducted in preterm infants born at <32 weeks' gestation and with BW of <1,500 g, and admitted to the neonatal intensive care unit (NICU) of Xinhua Hospital from December 2016 to 2017. The protocol was approved by the medical ethics committee of Xinhua Hospital, which is affiliated to Shanghai Jiao Tong University School of Medicine (XHEC-2016-139), and was registered on https://www.clinicaltrials.gov/ (NCT03100305). The exclusion criteria were as follows: infants with major or digestive congenital anomalies, infants with BW of ≥1.5 kg, and infants who transferred from the NICU before reaching total EN. GA was estimated based on obstetric records obtained during early-pregnancy ultrasound. Body weight was measured three times weekly using an electronic digital scale. The EUGR group was defined as infants with body weight below the 10th percentile for postmenstrual age, as plotted on the 2013 Fenton growth curves, when total EN is stable.

### Nutrition Management

Individualized PN was prescribed daily by nutritionists depending on infant metabolic status and medical criteria, validated by pharmacists, and prepared by nurses at the pharmacy department following good manufacturing practices. Guidelines for nutrition support in neonates in China (2013) were used in the neonatal unit. PN initiation was recommended within 24 h of birth at 80 ml/kg/d and increased by 20 ml/kg/d as infusion, containing dextrose infusion at a rate of 4–8 mg/kg/min; amino acids infusion (Pediatric Compound Amino Acid injection 18AA-II, PAA 6%; Treeful, Shanghai, China) initiated at 1.5–2 g/kg/d, and increased at a rate of 0.5–1.g/kg/d to the goal of 3.5–4.0 g/kg/d; and lipid infusion (Lipofundin MCT/LCT, 20%; Braun Medical, Melsungen, Germany) initiated at 1 g/kg/d, increased at a rate of 0.5–1 g/kg/d to the goal of 3 g/kg/d. Since daily PN was only available after 15:00, some newborns could not receive individualized PN within 24 h after birth.

Enteral feeding was initiated when the baby showed tolerance as soon as possible after birth. The mean human milk content was assumed to be 68 kcal/dL with 1.5 g/dL of protein. Various infant formula compositions provided by the manufacturers were listed in Table 1. To more accurately compare energy intakes between PN and EN, enteral caloric intakes were adjusted for predicted energy losses due to incomplete absorption and specific dynamic action, calculated at 85% of their original caloric values. Trophic enteral nutrition is defined as minimal volumes of milk feeds (<20 mL/kg/day), EN is defined as starting nutritional feeds above 20 mL/kg/day, and increasing to total EN feeds of 160–180 mL/kg/day.

**Table 1:** Nutritional compositions of human milk and infant formulas.

	**Protein** **(g/dl)**	**Energy** **(kcal/dl)**
Human milk	1.5	68
Preterm infant formula	2.3	80
Partially hydrolyzed formula	1.3	67
Extensively hydrolyzed formula	1.8/1.9	66/80
Amino acid based formula	1.8	67

### Sample Collection and Assays

Sampling point

Samples were collected dynamically at four sampling points:

1. Sampling point 1: within 24 h after birth;
2. Sampling point 2: when stable PN reached and before EN started, AA infusion in PN increased to the goal of 3.5–4.0 g/kg/d or be stable for 2–3 days;
3. Sampling point 3: EN energy intake reached 50% of the total energy intake;
4. Sampling point 4: full intestinal feeding was reached.

Then the nutrition regimens were divided into three phases from PN to EN based on the sampling point: (1) Phase 1: the total PN phase, which started from sampling point 1–2, (2) Phase 2: the early nutrition transitional phase, which started from sampling point 2–3, (3) Phase 3: the later nutrition transitional phase, which started from sampling point 3–4.

AA profiles were collected from the infants' heels as spots, which were dried at room temperature, stored at −20°C, and quantified using gas chromatography-mass spectrometry (GC-MS) with the Waters Xevo-TQ. The AA profiles included essential amino-acid (EAA): threonine (Thr) arginine (Arg), methionine (Met), phenylalanine (Phe), leucine (Leu), tryptophan (Trp) and valine (Val), as well as non-essential amino-acid (NEAA): citrulline (Cit), tyrosine (Tyr), proline (Pro), alanine (Ala), aspartic acid (Asp), glutamine (Gln), glutamate (Glu), glycine (Gly), histidine (His), ornithine (Orn), and serine (Ser). The internal isotope standard for tandem mass spectrometry was NSK-A from Cambridge Isotope Laboratories.

### Perinatal and Clinical Characteristics

Data for each eligible infant were collected: antenatal characteristics including steroid administration, preeclampsia, diabetes, and thyroid abnormalities; delivery history including sex, BW and its z-score, GA, Apgar score at 1 and 5 min; and clinical factors such as bronchopulmonary dysplasia (BPD; need for supplemental oxygen at 36 weeks' corrected GA), sepsis (confirmed by positive sampling point or urine culture), and severe intraventricular hemorrhage (IVH; if grade ≥3 and diagnosed by ultrasound), and pulmonary hypertension (PH).

### Statistics

The SPSS 22.0 (New York, NY) was performed as Statistical analysis. The normality analysis was used with Shapiro-Wilk test. The *t*-test or Mann-Whitney *U*-test was conducted for continuous variables, and the chi-square test was used for categorical variables. Spearman correlation was administered to reveal the association between AAs concentration with nutritional parameters. Multivariate regression analysis was used to validate the independent association of the AA concentrations with the risk of EUGR, and the nutritional parameters. Statistical significance was assumed at *p* < 0.05. Variables that reached statistical significance on univariate analysis for EUGR were included in the model.

## Results

**Figure 1:**
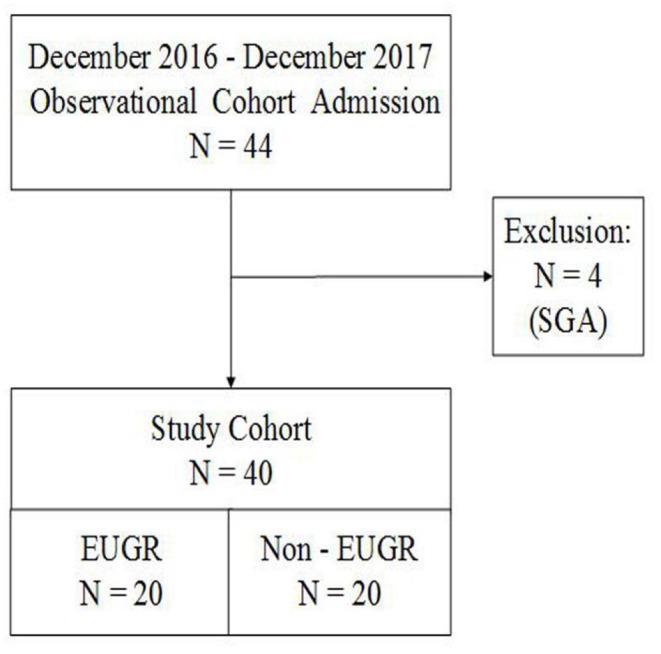
Flow chart of all preterm infants born with <32 weeks' gestation and BW of <1,500 g. AGA, appropriate for gestational age; BW, birth weight; EUGR, extrauterine growth retardation; GA, gestational age; SGA, small for gestational age.

A total of 44 preterm infants with a BW of <1,500 g and a GA of <32 weeks were consecutively selected for this registered observational cohort study. We excluded four small-for-gestational-age (SGA) infants with a BW < the 10th percentile. Finally, this study included 20 infants in the EUGR group and 20 infants in the non-EUGR group (Figure 1).

A distinctive difference in birth weight z-score was observed between both groups. Infants in the EUGR group had lower birth weight z-score than those in the non-EUGR group, with statistically significant difference (*p* < 0.05), even though there was no difference in gestational age and birth weight. There were no significant differences in sex, multiple birth, antenatal steroid use, complications of maternal pregnancy, and delivery history between groups. Postnatal age and actual weight of infants at sampling points 2, 3, and 4 were comparable between the two groups. Moreover, there was no significant difference in incidences of BPD, severe IVH, PH, and sepsis between both groups. The demographics and clinical factors are summarized in Table 2.

**Table 2:** Demographic and clinical factors in the Extrauterine Growth Retardation (EUGR) group and Non-EUGR group.

	**EUGR (*n* = 20)**	**Non-EUGR (*n* = 20)**		***p*-value**
**Demographics**				
Gestational age (*w*)	29.89 ± 1.90	28.93 ± 1.58	*t* = 1.72	0.09
Birth weight (*g*)	1188 ± 177	1287± 191	*t* = −1.69	0.09
Birth weight z-score	−0.53 ± 0.11	0.38 ± 0.12	*t* = −5.41	0.00^*^
Age of return to prior birth weight (*d*)	11.84 ± 1.67	10.85 ± 1.22	*t* = 0.48	0.63
Males (*n*)	12	12	χ^2^ = 0.00	1.00
Females (*n*)	8	8		
Asphyxia	13	17	χ^2^ = 1.20	0.27
	7	3		
Hypertension or eclampsia (*n*)	6	2	χ^2^ = 2.50	0.11
Diabetes or glucose abnormalities (*n*)	5	3	χ^2^ = 0.62	0.42
Thyroid abnormalities (*n*)	2	2	χ^2^ = 0.00	1.00
Postnatal corticosteroids (*n*)	2	1	χ^2^ = 0.36	0.54
Age at sampling point 2 (*d*)	5 (3, 8)	4 (2, 9)	*Z* = −1.30	0.19
Age at sampling point 3 (*d*)	16 (9, 51)	14 (9, 26)	*Z* = −1.01	0.30
Age at sampling point 4 (*d*)	29 (15, 58)	28 (15, 45)	*Z* = −0.84	0.40
Weight at sampling point 2 (*g*)	1,167 ± 179	1,231 ± 194	*t* = −1.08	0.28
Weight at sampling point 3 (*g*)	1,323 ± 174	1,354 ± 179	*t* = −0.55	0.58
Weight at sampling point 4 (*g*)	1,642 ± 221	1,706 ± 209	*t* = −0.94	0.35
Severe intraventricular hemorrhage (*n*)	2	3	χ^2^ = 0.04	0.82
Bronchopulmonary dysplasia (*n*)	7	6	χ^2^ = 0.11	0.73
Pulmonary hypertension (*n*)	1	2	χ^2^ = 0.36	0.54
Sepsis (*n*)	4	2	χ^2^ = 0.79	0.37

* *p < 0.05, significant difference between groups*.

### AA Profiles

**Figure 2:**
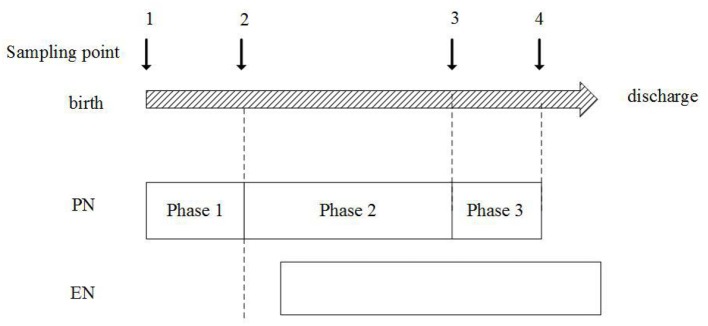
Schematic overview of study periods. PN, parenteral nutrition; EN, enteral nutrition.

**Figure 3:**
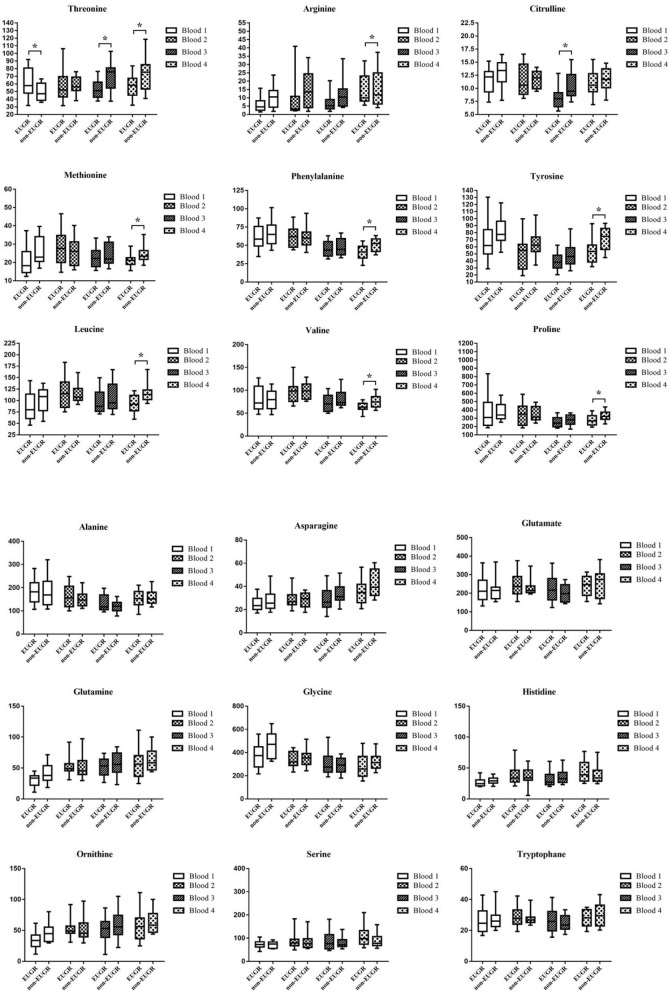
Box and whisker plots of 18 AAs (umol/L) at 4 blood sampling points. The box shows the medians (solid bar), interquartile ranges (box), and 90 and 10th percentiles (whiskers). *p < 0.05, significant difference between EUGR and non-EUGR groups.

Schematic overview of the study are exhibited in Figure 2. Meantime, the AA concentrations in dried sampling point spots (DBS) are shown in Figure 3. At sampling points 1 and 2, there was no significant difference in AA concentration between 2 groups, except Thr at point 1 which is higher in the EUGR group. At sampling point 3, lower Thr and Cit were detected in the EUGR group. As weaning off PN toward total EN, AA deficiency progressively emerged in the EUGR group compared with AA in the non-EUGR group, especially for most EAAs: Thr, Arg, Met, Phe, Leu, and Val. Moreover, the NEAAs including Cit, Tyr and pro were lower in the EUGR group (Table 3 and Figure 3).

**Table 3:** Amino acids in the Extrauterine Growth Retardation (EUGR) group and Non-EUGR group.

**EUGR vs. non-EUGR**			***p*-value**
Thr at sampling point 1	75.8 (37.3, 102.5)	58.0 (34.4, 98.8)	0.023^*^
Arg at sampling point 3	8.1 (1.8, 21.3)	14.0 (4.1, 33.4)	0.017^*^
Cit at sampling point 3	8.4 (5.8, 12.1)	10.5 (7.4, 15.5)	0.008^*^
Thr at sampling point 3	57.4 (37.6, 80.3)	69.5 (37.1, 103.5)	0.041^*^
Met at sampling point 4	20.9 (14.1, 27.1)	25.0 (18.4, 35.3)	0.020^*^
Phe at sampling point 4	41.5 (23.6, 50.8)	50.7 (37.1, 63.3)	0.014^*^
Tyr at sampling point 4	55.8 (33.1, 91.7)	71.8 (44.7, 93.3)	0.017^*^
Leu at sampling point 4	94.6 (62.0, 120.8)	116.7 (93.5, 167.7)	0.005^*^
Val at sampling point 4	65.1 (46.0, 79.8)	77.0 (56.4, 101.9)	0.028^*^
Thr at sampling point 4	57.5 (33.8, 81.6)	73.0 (40.9, 118.4)	0.040^*^
Pro at sampling point 4	277.6 (196.3, 379.4)	330.0 (231.7, 435.0)	0.020^*^

Arg, arginine; Cit, citrulline; Leu, leucine; Met, methionine; Phe, phenylalanine; Thr, threonine; Tyr, tyrosine; Val, valine; Pro, proline.

* *p < 0.05, significant difference between groups*.

### Associations of AA Concentrations With EUGR

Associations between the AAs level with the risk of EUGR were analyzed using multivariate regression analysis. When the above AA concentrations were corrected for GA and birth weight z-score, Cit at sampling point 3, Thr at sampling point 3, Met at sampling point 4, and Phe at sampling point 4 were found to be independent predictors of EUGR (Table 4).

**Table 4:** Multivariate analysis of predictors of extrauterine growth retardation.

**Predictors of postnatal growth failure**	**Odds ratio**	**95% CI**
Thr at sampling point 1	0.94	0.86–1.02
Arg at sampling point 3	1.19	0.99–1.44
Cit at sampling point 3	1.62	1.03–2.56
Thr at sampling point 3	1.06	1.01–1.12
Met at sampling point 4	1.15	0.91–1.47
Phe at sampling point 4	1.06	0.95–1.18
Tyr at sampling point 4	1.05	0.98–1.12
Leu at sampling point 4	1.16	0.99–1.37
Val at sampling point 4	1.16	1.02–1.31
Thr at sampling point 4	1.05	0.99–1.11
Pro at sampling point 4	1.06	1.01–1.12

*Arg, arginine; CI, confidence interval; Cit, citrulline; Leu, leucine; Met, methionine; Phe, phenylalanine; Thr, threonine; Tyr, tyrosine; Val, valine; Pro, proline*.

### Nutrition Intakes

**Figure 4:**
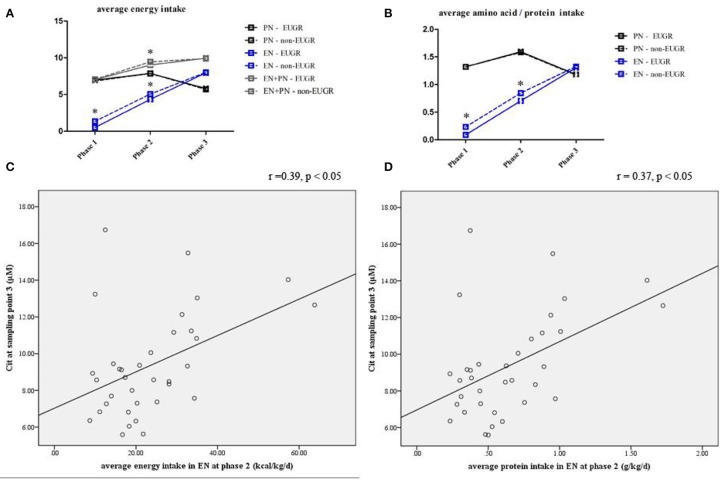
Line chart of energy and protein intakes during different nutritional phases in the EUGR and non-EUGR groups **(A,B)**. The box shows the median ± SD.*p < 0.05, significantly higher vs. lower intakes (*p* < 0.05). Correlation between Cit concentration with average energy (*r* = 0.39, *p* < 0.05) and protein intakes (*r* = 0.37, *p* < 0.05) in EN at the corresponding nutritional phases **(C,D)**.

Nutritional intakes at the three nutritional phases were calculated for each infant (Table 5). Infants in the EUGR group started trophic EN and EN later than those in the non-EUGR group. Infants in the EUGR group had lower average intakes of AAs and energy in EN at phase 1 and non-distinctive average intakes of AAs and energy in PN, compared with average intakes in the non-EUGR group. During the weaning of PN, infants in the EUGR group had lower average intakes of AAs and energy in EN at phase 2 (*p* = 0.03 and *p* = 0.04, respectively). Besides, there were no significant differences in age when PN was started, in average intakes of AAs, or in energy in PN at all three phases (Table 5). Charts were employed to show dynamic changes in AAs and energy intakes from PN to EN, provided that infants in the EUGR group have lower protein and energy intakes in EN in the early transitional phase (Figures 4A,B).

**Table 5:** Nutrition parameters and sampling point Urea Nitrogen (BUN) concentrations in the Extrauterine Growth Retardation (EUGR) group and Non-EUGR group.

	**EUGR**	**Non-EUGR**	***t* / *Z***	***p***
Age when PN was started (*d*)	1.05 (1, 1.9)	0.84 (0, 2)	*Z* = −1.547	0.12
Age when trophic EN was started (*d*)	6.75 (2.1, 12.9)	4.31 (1, 10)	*Z* = −2.05	0.04^*^
Age when EN was started (*d*)	9.2 (4, 17.6)	6.8 (3, 13)	*Z* = −1.80	0.07
Duration between sampling point 2 and sampling point 3 (*d*)	14 (4.2, 35)	10.4 (5, 19)	*Z* = −1.07	0.28
Duration between sampling point 3 and sampling point 4 (*d*)	14.15 (7.1, 29)	12.6 (5, 25)	*Z* = −0.53	0.59
**Phase 1**				
Average amino acid concentration in PN (g/kg/d)	1.76 ± 0.45	1.75 ± 0.28	*t* = 0.11	0.91
Average energy in PN (kcal/kg/d)	48.91 ± 10.01	47.14 ± 7.38	*t* = 0.62	0.53
Average amino acid concentration in EN (g/kg/d)	0.02 (0, 0.08)	0.1 (0, 0.37)	*Z* = −2.27	0.02^*^
Average energy in EN (kcal/kg/d)	0.84 (0, 2.52)	3.27 (0, 12.29)	*Z* = −2.27	0.02^*^
Average energy in PN + EN (kcal/kg/d)	49.76 ± 9.75	50.42 ± 9.07	*t* = −0.22	0.82
**Phase 2**				
Average amino acid concentration in PN (g/kg/d)	2.55 ± 0.42	2.52± 0.41	*t* = 0.27	0.78
Average energy in PN (kcal/kg/d)	62.10 ± 7.98	62.06 ± 10.39	*t* = 0.01	0.99
Average amino acid concentration in EN (g/kg/d)	0.51 (0.23, 0.87)	0.75 (0.30, 1.61)	*Z* = −2.04	0.03^*^
Average energy in EN (kcal/kg/d)	19.52 ± 7.24	27.16 ± 14.42	*Z* = −2.07	0.04^*^
Average energy in PN + EN (kcal/kg/d)	81.62 ± 8.43	89.23 ± 9.01	*t* = −2.72	0.01^*^
**Phase 3**				
Average amino acid concentration in PN (g/kg/d)	1.41 (0.88, 2.05)	1.38 (1.03, 1.79)	*Z* = −0.47	0.63
Average energy in PN (kcal/kg/d)	34.43 (21.44, 54.45)	32.90 (25.37, 46.01)	*Z* = −0.81	0.41
Average amino acid concentration in EN (g/kg/d)	1.74 (0.82, 2.25)	1.80 (0.99, 2.23)	*Z* = −0.30	0.75
Average energy in EN (kcal/kg/d)	64.82 (28.60, 81.17)	65.38 (45.19, 82.91)	*Z* = −0.19	0.84
Average energy in PN + EN (kcal/kg/d)	99.25 ± 11.45	98.29 ± 12.70	*t* = 0.27	0.80

EN, enteral nutrition; PN, parenteral nutrition; TEN, total enteral nutrition.

* *p < 0.05, significant difference between groups*.

### Associations of AA Concentrations With Nutritional Parameters

Spearman analysis showed a positive correlation between the Cit level at sampling point 3 and average energy intake (*r* = 0.39, *p* = 0.02; Figure 4C) and protein intake (*r* = 0.37, *p* = 0.03; Figure 4D) in EN during the early transitional phase, respectively. Multivariate linear regression showed that the association between Cit concentration at sampling point 3 and average energy and protein intake in EN at phase 2 remained significant, after adjusted for GA and BW z-score (*r*^2^ = 0.17, *p* = 0.02 and *r*^2^ = 0.15, *p* = 0.03, respectively).

### Power Analysis

*Post-hoc* power calculation was performed based on the natural logarithmic citrulline concentration, using PASS 2020 software (NCSS, LCC). Given a power of 0.80 and a two-tailed alpha error probability of 0.05, 15 infants in each group is necessary to obtain significant difference between the two groups.

## Discussion

This study described the AA metabolomic profiles of preterm infants during different nutritional phases. Based on the findings, concentrations of Cit and Thr at sampling point 3 and Met and Pro at sampling point 4 were compromised in the EUGR group and were associated with a higher risk for EUGR when full EN started. Furthermore, a deficiency in Cit concentration at sampling point 3 was positively correlated with energy deficit and, protein deficit in EN during the early transitional phase.

Due to immaturity and intolerance, most preterm infants are indicated for initial PN and subsequent enteral feedings which are frequently interrupted. To examine the association between AA metabolic patterns and nutrition regimens during the nutritional transition, we used neonatal data by nutritional phases as described by Miller et al. (14), but rather chronologically (8, 15–17). This method better identifies the correlation of AA metabolites with nutrition, as well as the phases across the nutritional therapy spectrum during which preterm infants are most vulnerable to nutrition deficit (13), and allows for practical and optimal nutrition management for preterm infants.

In this study, there was no significant difference in AA patterns at birth between the two groups, except for Thr, which was higher at sampling point 1 and had lower concentration in the EUGR group at sampling points 3 and 4.The high Thr level after birth soon may be related to mother' metabolic condition, It hadn't been reported before that Thr was higher after birth in EUGR infants yet. Moreover, threonine kinetics studies indicated a high obligatory visceral need for threonine, presumably for the purposes of synthesis (18). The higher Thr after birth in the EUGR group maybe involved in high rate of metabolism. Meanwhile, Other studies in preterm infants also indicated that the Thr content of the current neonatal PN is too high (19), and intravenous infusion may directly increase the Thr concentration. Dual stable-isotope tracer techniques suggested that the intestine was the major site for uptake and utilization of Thr (18). The restriction of enteral feeding during transition may give rise to a Thr deficiency in the EUGR group. In turn, the Thr deficiency may impair the synthesis of crucial mucosal cellular proteins and the maturation of intestine. Cit, a non-essential AA that is not incorporated into a protein, is produced in the small intestine and participate in the urea nitrogen cycle (20); It has been shown that the enterocytes are responsible of Cit synthesis (21). Intestinal epithelial injury can cause reduced Cit production as shown in necrotizing enterocolitis (NEC), short bowel syndrome and numerous other intestinal disease states (22–25), which indicated that Cit levels may be correlated with functional intestinal mass potentially The lower Cit concentration during transition may suggest the intestine is relatively immature in the EUGR group compared with non-EUGR group.

Previous studies have raised concerns about the effect of AAs on growth and development, but such effect remains inconsistent and undefined. In this study, the multivariate regression analysis showed the correlation of lower concentrations of Cit and Thr at sampling point 3 as well as Met and Phe at sampling point 4 with the high risk of EUGR after adjusted for GA, birth weight z-score, age when trophic EN was started, as well as average energy and protein intakes in synchronous nutritional period. Of particular interest was Cit and Thr at sampling point 3, which occurred ahead of sampling point 4 and could effectively predict EUGR in advance. Animal studies have raised concerns about the effect of hyperthreoninemia on the developing brain, however, its effect on growth is blurry. Neonatal Cit administration would enhance growth and prevent the metabolic consequences of intrauterine growth retardation through an “imprinting” effect on the liver lipidome and fat metabolism (20, 26). We examined contemporaneous nutrition intakes during the transitional phase and found that infants in the EUGR group had protein and energy deficits in EN during phase 1 and the early transitional phase (phase 2), compared with infants in the non-EUGR group. This finding is consistent with previous observations, wherein both protein and energy deficits were responsible for poor growth during transition from PN to total EN (14). Additionally, protein intake is a much stronger factor for growth than total energy intake (14, 27). Our study further analyzed the association between protein intake and AA pattern. Multivariate regression analysis revealed that the Cit level during early transition was correlated to average energy and protein intake in synchronous nutritional period after corrected for GA and birth weight Z-score. Studies have found that the Cit levels were positively correlated with the rate of enteral advancement (28). The correlation of Cit levels along with the nutritional intakes in EN suggests that Cit level may be a sensitive marker of intestinal function. The lower Cit concentration during transition may suggest the intestine is relatively immature in the EUGR group compared with non-EUGR group. However, it is difficult to distinguish the causal relationship between the low Cit concentration and the immaturity of intestine.

Our study has some limitations. First, it has a observational design. However, to better examine the associations between metabolism, growth, and contemporaneous nutrition regimens, we defined EUGR as weight below the 10th percentile, when total EN is stable, rather than discharge commonly used. Second, time frames for growth rates were not included in this study. Lastly, although there were no differences in major medical conditions between the two groups, all nutritional phases may not have been excluded uniformly, creating a potential bias. No consensus exists for a normative AA profile in preterm infants, which may be related to the different immaturity, clinical status, and nutritional support and the like. Studies have revealed a dynamic fluctuation in AA patterns, but it is not yet to determine whether it is physiological or not. Previous studies have revealed different factors that influenced the AA profiles, including mothers' condition, GA, BW, age of sampling, and disease status, etc. More studies are needed to further reveal the metabolism of AAs in preterm infants.

In conclusion, we found that a lower Cit concentration during the transitional phase was associated with an increased likelihood of EUGR in infants, and positively correlated with contemporaneous nutrition intake. It should prompt the clinician involved in neonatal nutrition management to more closely examine nutrition practices to prevent the incidence of EUGR. More aggressive PN management during the early transitional phase could be encouraged to minimize nutrient deficit. Furthermore, the follow-up study is necessary about AA metabolic patterns on the long-term growth and development.

## Data Availability Statement

The datasets generated for this study are available on request to the corresponding author.

## Ethics Statement

The studies involving human participants were reviewed and approved by the medical ethics committee of Xinhua Hospital, which is affiliated to Shanghai Jiao Tong University School of Medicine (XHEC-2016-139). Written informed consent to participate in this study was provided by the participants' legal guardian/next of kin.

## Author Contributions

ZH, YW, and LH contributed to the conception and design of the research. DL, LW, and HS contributed to the acquisition of the specimen and data. LW contributed to the analysis, and interpretation of the data. All authors drafted the manuscript, critically revised the manuscript, agreed to be fully accountable for ensuring the integrity and accuracy of the work, and read and approved the final manuscript.

## Conflict of Interest

The authors declare that the research was conducted in the absence of any commercial or financial relationships that could be construed as a potential conflict of interest.
